# Pancreatic Ductal Adenocarcinoma: Relating Biomechanics and Prognosis

**DOI:** 10.3390/jcm10122711

**Published:** 2021-06-19

**Authors:** Benjamin M. MacCurtain, Ned P. Quirke, Stephen D. Thorpe, Tom K. Gallagher

**Affiliations:** 1 Department of Hepatobiliary and Transplant Surgery, St Vincent’s University Hospital, D04 T6F4 Dublin, Ireland; benjaminmaccurtain@rcsi.com; 2 UCD School of Medicine, University College Dublin, D04 V1W8 Dublin, Ireland; ned.quirke@ucdconnect.ie (N.P.Q.); stephen.thorpe@ucd.ie (S.D.T.); 3 UCD Conway Institute of Biomolecular & Biomedical Research, University College Dublin, D04 V1W8 Dublin, Ireland

**Keywords:** pancreatic cancer, pancreatic ductal adenocarcinoma (PDAC), neoadjuvant therapy, biomechanics, tissue mechanics, tumour microenvironment, extracellular matrix (ECM), elastography, endoscopic ultrasound, magnetic resonance elastography

## Abstract

Pancreatic ductal adenocarcinoma (PDAC) is the most common form of pancreatic cancer and carries a dismal prognosis. Resectable patients are treated predominantly with surgery while borderline resectable patients may receive neoadjuvant treatment (NAT) to downstage their disease prior to possible resection. PDAC tissue is stiffer than healthy pancreas, and tissue stiffness is associated with cancer progression. Another feature of PDAC is increased tissue heterogeneity. We postulate that tumour stiffness and heterogeneity may be used alongside currently employed diagnostics to better predict prognosis and response to treatment. In this review we summarise the biomechanical changes observed in PDAC, explore the factors behind these changes and describe the clinical consequences. We identify methods available for assessing PDAC biomechanics ex vivo and in vivo, outlining the relative merits of each. Finally, we discuss the potential use of radiological imaging for prognostic use.

## 1. Introduction

Pancreatic cancer is the fourth leading cause of cancer related mortality in the US and Europe [1,2], and is projected to become the second leading cause by 2030 [3]. Pancreatic ductal adenocarcinoma (PDAC) is the most common form of pancreatic cancer and accounts for up to 90% of pancreatic neoplasms [4]. Despite being relatively rare with reported incidence in US and Europe in the range of 11.5–15.3 per 100,000 [2,5,6], five-year survival is poor and consistently less than 10% [1,7]. There are several reasons for this poor prognosis. Symptoms are often non-specific leading to diagnosis only when the cancer is at an advanced stage. Furthermore, it exhibits a remarkable resistance to conventional treatment options including chemotherapy, radiotherapy, and immunotherapy [8,9].

This leaves surgery as the only potentially curative option, and for the 10–20% of patients who undergo surgical resection, the five-year survival rate is 15–25% [10]. Adjuvant therapy with a modified FOLFIRINOX regime has recently been shown to extend median overall survival to 54.4 months for patients postresection, with 3-year survival rates of 63.4% [11]. For the approximately 80% of patients who present with locally advanced or metastatic disease, gemcitabine or 5-fluorouracil chemotherapy provides survival times of the order of months, with some slight improvements using nab-paclitaxel with gemcitabine, or FOLFIRINOX [12,13]. Over the past decade, much of the clinical attention has focussed on converting those who present with locally advanced or borderline resectable disease to a resectable status using neoadjuvant chemotherapy (NAT) [14], and radiation therapy [15,16,17,18].

**Figure 1:**
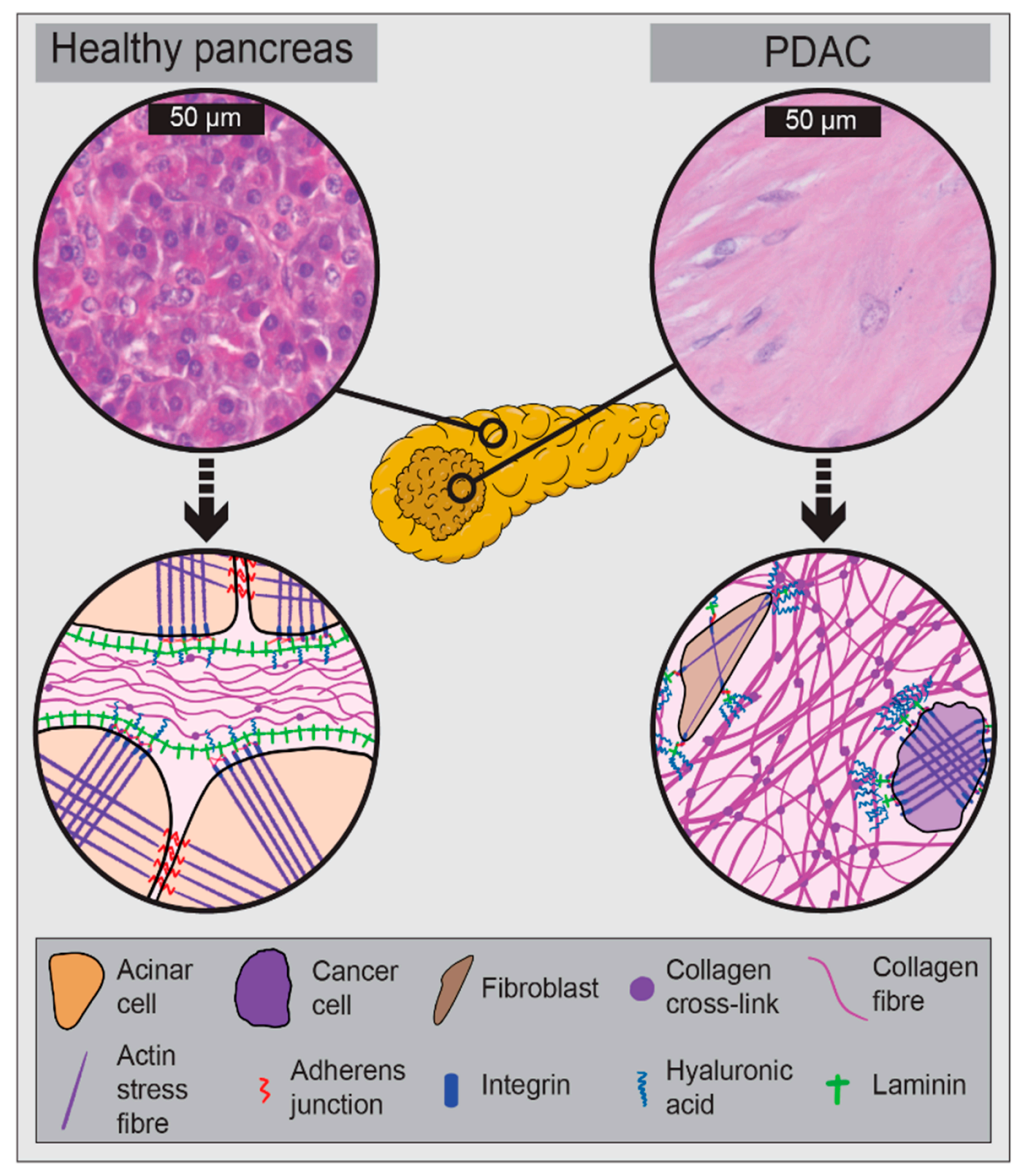
Schematic illustration of tumour changes from healthy pancreas to pancreatic ductal adenocarcinoma (PDAC). Top: Haematoxylin and eosin (H&E) staining of healthy human pancreas and resected PDAC tissue. Bottom: Schematic representation of cell–cell and cell–extracellular matrix (ECM) interactions in healthy pancreas and PDAC. PDAC is associated with increases in stromal volume, collagen fibre thickness, collagen crosslink density, hyaluronic acid, reduced laminin, and a switch from cell–cell connections to integrin mediated cell-ECM connections providing a bridge between the contractile cytoskeleton and the ECM.

PDAC is characterised by the formation of a dense desmoplastic reaction (stroma) surrounding malignant epithelial cells of ductal origin (Figure 1). This stromal component of PDAC accounts for up to 80% of the tumour mass [19], and is responsible for making PDAC one of the stiffest malignancies with solid stress values approaching 10 kPa [20,21]. Indeed, the physical properties of the tumour and stroma have been shown to be several magnitudes stiffer than healthy pancreatic tissue [22], and there is a well-documented link between stromal stiffness and cancer progression [23,24]. It is likely that stromal stiffness reflects and influences clinically relevant outcomes. We hypothesise that stromal stiffness may serve as a biomarker both in PDAC diagnosis and in the evaluation of treatment response.

The aims of this review are threefold: (i) to describe the biomechanical changes seen on a tissue and cellular level in PDAC; (ii) to critically evaluate diagnostic and prognostic implications for the study of PDAC biomechanics with a focus on the relationship of tumour and stroma stiffness to oncogenesis, therapy and chemoresistance; and (iii) to explore the in vitro and in vivo methods available for the assessment of PDAC biomechanics. Recommendations regarding future work and how this may benefit patients are proposed.

## 2. The Biomechanical PDAC Microenvironment

Biomechanics is the study of the mechanical aspects of biological systems and integrates material properties with structure and function (Table 1). The biomechanical properties of a tissue are a function of cellular and extracellular matrix (ECM) properties. Due to tissue component turn over, tissue mechanics can be dynamic. Indeed, in desmoplastic and fibrotic scenarios such as PDAC, tissue mechanics evolve toward progressively increased stiffness and solid stress [25]. This is ultimately driven by the cellular component of the tissue which is mechanically continuous with the ECM. PDAC tissue can be described as a viscoelastic material as it exhibits both solid and fluid behaviour, with fluid pressure relaxation under load [26,27].

The primary biomechanical change seen in PDAC is an increase in fibrosis and thus stiffness [27]. This has been described using both in vivo and in vitro methods, consistently demonstrating that pancreatic tumours are some magnitudes stiffer than normal or inflamed pancreatic tissue [27,28,29]. One study used atomic force microscopy (AFM) to assess the stiffness of healthy mouse pancreas, pancreatic intraepithelial neoplasia (PanIN), and PDAC [30]. Healthy mouse pancreas exhibited a Young’s modulus of approx. 500 Pa, while the average stiffness increased 2- and 3-fold in in PanIN and PDAC, respectively. In addition to overall increases in stiffness, the heterogeneity in tissue structure that develops with PDAC leads to much greater localised increases in Young’s modulus with regional values approaching 10 kPa [30]. This increase in stiffness is achieved through the production of ECM proteins which also increase interstitial hydrodynamic pressure [26,31]. PDAC tissue has also been shown to possess anisotropic properties so that its compliance is dependent on the direction of applied force [32], which should be considered when interpreting biomechanical data from PDAC tissue.

### 2.1. Drivers of PDAC Biomechanics

The elaboration of an extensive ECM is the key driver of increased stiffness in PDAC. Ordinarily quiescent pancreatic stellate cells (PSCs) are recruited by cancer cells through paracrine signalling and become activated, forming much of the cancer associated fibroblast (CAF) population in PDAC [33]. Factors including matrix stiffness and an acidic tumour microenvironment further contribute to the activation of PSCs [34,35]. While quiescent PSCs secrete few proteins, activated PSCs secrete a complex mixture of proteins with important roles in wound healing, inflammation, cancer cell proliferation, inhibition of apoptosis, fibrosis and invasion [36]. In PDAC, cancer cells drive chronic activation of PSCs through paracrine signalling involving platelet derived growth factor (PDGF) which drives PSC proliferation, and both transforming growth factor β-1 (TGF-β-1) and fibroblast growth factor 2 (FGF-2) which drive elaboration of an ECM rich in fibronectin and collagens I, III, and IV [37,38,39]. The most abundant ECM component in PDAC is collagen type I [40,41]. Another constituent of the ECM upregulated in PDAC is the glycosaminoglycan hyaluronan [42], which has been shown to increase cancer cell motility [43,44], and is associated with an increased incidence of metastasis [45]. Increased levels of both collagen type I and hyaluronic acid within the stroma have been associated with reduced patient survival [46], although as indicated above, the stroma may act to both restrain and encourage PDAC progression.

In addition to matrix abundance, crosslinking of matrix components also drives tissue mechanics. Drivers of collagen crosslinking in pancreatic cancer include the enzymes lysyl oxidase (LOX) and transglutaminase-2 (TGM-2). LOX is a copper dependent enzyme that crosslinks collagen and elastin, and inhibition of LOX has been shown to suppress metastasis in mouse models [47]. The PDAC tumour microenvironment is hypovascular and as a result is hypoxic. Hypoxia, through activation of hypoxia inducible factor-1α (HIF-1α), drives expression of LOX [47] to increase tumour stiffness [48]. TGM-2 has both intracellular and extracellular functions. It can induce focal adhesion kinase (FAK) signalling to drive invasiveness and drug resistance [49,50]. TGM-2 is also secreted from cells where it crosslinks and stabilises the ECM making it resistant to both mechanical and proteolytic perturbation [51].

Proteolytic enzymes also play a role in the biomechanics of PDAC. One key collagenase, membrane-type-1 matrix metalloproteinase (MT1-MMP), is upregulated in response to high collagen content in PDAC [52]. MT1-MMP is a membrane-anchored MMP used by stromal and cancer cells to degrade fibrillar collagen [53], facilitating fibre realignment and creation of ECM tracks for cancer cell dissemination [54]. While MT1-MMP expression is driven by TGF-β-1 signalling, MT1-MMP also contributes to fibrosis by stimulating further TGF-β signalling and resultant collagen production [55].

Cells respond to biomechanical changes in their environment, such as increased stiffness, by changing their phenotype through a process called mechanotransduction [56]. This can lead to a positive feedback loop whereby increasing tissue stiffness through increased cellular tension drives further increases in tissue elaboration and remodelling [57]. The evolution of mechanical anisotropy is a result of remodelling and correlates with CAF shape and orientation [32]. Development of ECM anisotropy in other cancers has been shown to involve mechanotransduction via cell-ECM interactions, and is associated with tumour invasion and metastasis [58,59]. Indeed, the association between increased ECM stiffness and cellular invasion is conserved across fibrotic tissues [60]. Several mechanotransduction signalling pathways may be involved in driving matrix production and ultimately tumour progression and have been recently reviewed by Broders-Bondon et al. [61].

While the extensive ECM deposition in PDAC has been linked with poor treatment outcomes, targeting the ECM has not been successful to date [62]. Indeed, the stroma has been shown to restrain PDAC progression [63]. Recent proteomic analysis of stromal composition reveals that while both cancer cells and CAFs contribute to stromal matrix elaboration, tumour cell derived proteins were correlated with poor patient survival and metastasis [64,65]. This suggests that more precise stromal targeting strategies focussed on tumour-promoting proteins may be more effective, and is an area which warrants further study [66]. In summary, ECM constituents including collagen and hyaluronan are altered in PDAC and contribute to tumour progression. Some of the described drivers of PDAC biomechanics are illustrated in Figure 1.

### 2.2. Consequences of PDAC Biomechanics

#### 2.2.1. Increased Invasiveness

There is a causal link between PDAC stiffness and increased tumour invasive potential [20,23,67]. Some postulated mechanisms of this increased invasiveness include increased expression of nuclear mechanoregulatory protein lamin A [20], as well as growth factor receptor signalling, cell junction disruption, thickening of collagen fibres, collagen type I driven EMT and increased cell migration [23,67]. Increased cell alignment in PDAC has been associated with increasing malignant potential of the tumour [30,68]. All relate poorer outcomes to tumour stiffness. Tension within tissue encourages cell migration from soft to stiff regions, a phenomenon coined durotaxis [69], which may have ramifications for PDAC metastasis [70]. Collagen type I activates focal adhesion kinase (FAK) signalling [71], while both collagen type I and hyaluronic acid promote epithelial-to-mesenchymal transition (EMT) leading to increased cancer stem cell (CSC) incidence, tumour growth and invasion [72,73,74].

#### 2.2.2. Decreased Drug Delivery

The mechanism of decreased drug delivery has been postulated to be due to vasculature compression which is related to a high tissue shear modulus and solid stress [75,76,77]. Jacobetz et al. demonstrate that hyaluronic acid impairs vascular function in a mouse model of PDAC [78], likely via hydraulic swelling within the PDAC stroma [26]. As stated above, tumour stiffness is positively associated with hyaluronic acid levels and poorer outcomes [42,46].

#### 2.2.3. Chemoresistance

PDAC treatment success may be limited by its inherent biomechanical properties. PSCs are the key drivers of PDAC biomechanics and remain active even under radiotherapy where they continue provide a tumour-supportive microenvironment [79], emphasising the potential influence of stiffness on reducing treatment options. The reduction of PSC activity has been reported to slow PDAC growth and improve survival in a mouse model [80], further bolstering this point. Increasing PDAC stiffness has been shown to support paclitaxel resistance [30], further illustrating the role of biomechanics as a contributor to poorer outcomes [57].

### 2.3. Neoadjuvant Therapy and PDAC Biomechanics

PDAC in a clinical context may be divided into resectable, borderline resectable, locally advanced unresectable and metastatic disease. The first three of these are defined by the tumour relationship to surrounding vasculature [81]. The goal of neoadjuvant treatment (NAT) in borderline resectable tumours is to allow downstaging to facilitate resection [18,82], and a number of trials have assessed the role of both chemotherapy and chemoradiotherapy based NAT in upfront resectable disease [83,84,85], the theory being that we would identify during treatment, those who are likely to progress rapidly and thus avoid an unnecessary and potentially morbid procedure for these patients [15,16,17]. Questions remain about the appropriate use of NAT in terms of what scenarios it should be used, for how long, and if radiotherapy has a role [86,87]. It has been observed that these positive outcomes may be due to tumour downstaging [88]. However, re-staging post NAT is difficult due to the lack of distinction between necrotic tumour and active cancer [82]. On conventional radiology, it is impossible to properly assess tumour response, to the point where most societies now acknowledge that the diagnostic performance of imaging studies is not sufficient to ensure the accurate selection of patients in whom negative-margin resection is likely to be achieved. More specifically, standard criteria for predicting vascular invasion, based on the amount of tumour-vessel contact, are not valid after NAT [89].

A potential avenue of investigation is that biomechanics of the tumour may change because of NAT and may be further impacted by the type of therapy, chemotherapy, radiotherapy or chemoradiotherapy. Alvarez et al. demonstrated a reduction in tumour stiffness when measured in vivo using endoscopic ultrasound elastography (EUSE) post NAT with two cycles of nab-paclitaxel and gemcitabine [90]. However, ex vivo analysis using harmonic motion elastography did not find a significant change in PDAC stiffness post NAT, although 11 different NAT regimes involving chemotherapeutics and radiotherapy were used within the 14 NAT samples [91]. In breast cancer, it has been shown that reductions in the stiffness of the tumour are positively correlated to response [92,93,94]. This further supports the call to incorporate biological and/or biomechanical rather than purely anatomical criteria for resectability [95], of which, stiffness may provide part of the solution. There may be a role therefore for the in vitro testing of human PDAC biopsy samples to characterise the relationship between stiffness and neoadjuvant therapy and correlate this to clinical outcome and post-resection specimens.

## 3. Ex Vivo Assessment of PDAC Biomechanics

**Figure 2:**
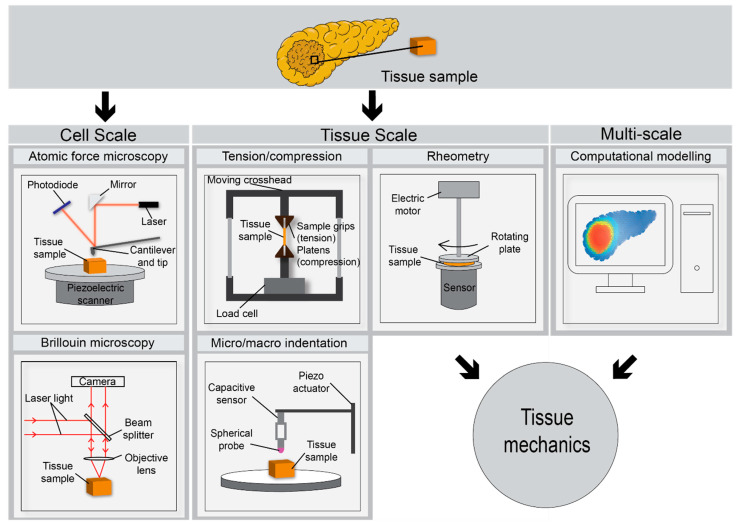
Schematic describing various methods for the ex vivo biochemical assessment of surgically resected PDAC tissue. The pancreatic sample is illustrated within each method as an amber coloured cube or rectangle. Atomic force microscopy (AFM) and Brillouin microscopy facilitate mechanical assessment on the nano-to-micro (nm-μm) scale relevant to cell-ECM interactions. Tensile and compressive testing, indentation and rheometry facilitate assessment of specimen mechanical properties on the macro (mm) scale. Computational modelling such as finite element analysis (FEA) can serve as an adjunct, filling in gaps in our knowledge based on extrapolated stiffness data at various length scales.

Several methods have been used in the mechanical testing of pancreatic tissue, and other biological samples (Figure 2). Pancreatic tissue derived from mice as well as surgical PDAC specimens have been successfully tested using atomic force microscopy (AFM) [30,57,63,96,97]. As the micro scale tip indents the specimen, the cantilever beam flexes as a function of the sample stiffness. As such, surface characteristics and biomechanical properties of the specimen can be measured by combining the tip position, cantilever spring constant and measurements made by a piezoelectric sensor [98]. The so called “force spectroscopy” mode is used to produce force-indentation curves, where the sample is moved towards the tip. High speed and multi-frequency AFM have recently enabled more detailed dynamic analyses on the nanoscale [98]. A diagrammatic representation of an AFM is shown in Figure 2. AFM facilitates assessment of both elastic and viscoelastic material properties, and such characteristics can aid in the differentiation between malignant and benign tissues [99].

Tensile and compression testing have also been employed to characterise soft biological samples [100,101]. The viscoelastic properties of human and porcine pancreata have also been tested using rheometer and plate systems [102,103]. Human and porcine pancreatic tissue were seen to behave similarly in terms of their viscoelastic properties. However, these were not PDAC samples [102], although this does indicate the possible validity of equating porcine pancreas biomechanics to that of humans. Viscoelastic properties were also observed to change with time post resection [103], reflecting a potential logistics issue for the ex vivo study of cancer tissue mechanics. This dynamic testing procedure is performed under compressive load while applying a shear stress to the specimen. It has been used to measure the stress as a function of time and axial deformation, Young’s and viscous modulus as a function of axial strain and finally, shear strain, phase shift (a product of the specimen being viscoelastic) and axial stress as a function of shear strain [104]. Furthermore, it has been proposed as a complimentary procedure to standard histology in colon cancer diagnosis [104].

These modalities measure gross tissue properties in a relatively rapid manner (several minutes) but do not account for the regional heterogeneity present in PDAC tissue. Mechanical assessments on shorter length scales are desired to assess tissue heterogeneity. Rubiano et al. used mesoscale indentation testing (1–4 mm diameter tip; Figure 2) to characterise the stiffness and viscoelastic properties of normal, pancreatitis and PDAC tissue [27]. Custom indentation apparatus was used to obtain viscoelastic properties. Both steady state modulus and viscosity were increased in PDAC compared to normal tissue containing no evidence of pancreatitis or malignancy [27]. AFM applies similar principles to indentation testing of tissue, but at a much shorter length scale which is dependent on the tip size which can range from tens of nm to several μm (e.g., 20 nm to 5 μm tip radius. Figure 2). However, with greater spatial resolution comes greatly reduced sample throughput which may the diagnostic potential of techniques such as AFM.

Brillouin microscopy is a method used to measure stiffness and viscoelastic properties through the material’s p-wave modulus in the GHz frequency range [105,106]. This non-contact 3D method of measurement works on the premise of photons (e.g., from a laser source) exciting microscopic acoustic waves in the material of interest (Figure 2). Mechanics of the sample are encoded in the frequency shift and linewidth of the Brillouin peaks which are collected on a camera. These displaced waves can then be measured to determine elastic and viscous components, respectively. This method provides resolution of approximately 1 μm and has shown promise in the mechanical assessment of both cells and heterogenous tissues [105]. It should be noted that at this method measures the p-wave or longitudinal modulus and not the Young’s modulus, the values can be several orders of magnitude different to classical measurement techniques [107].

Finite element analysis (FEA) is a useful complimentary approach to the characterisation of human tissue. It uses computational methods to predict the mechanical response or extrapolate the response based on experimental data [108,109] (Figure 2). Some advantages of this method include the prediction of biomechanical properties of PDAC based on previous experimental study data and the ability to bridge length scales between different mechanical testing modalities with relevance to the stiffness gradients that develop around tumour glands which can drive metastasis. It also has the potential to play a role in diagnosis whereby patient-specific clinical image data could provide boundary conditions for pre-characterised mechanical models of PDAC tissue to provide an indication of tissue stiffness.

## 4. In Vivo and Radiological Assessment of PDAC Biomechanics

**Figure 3:**
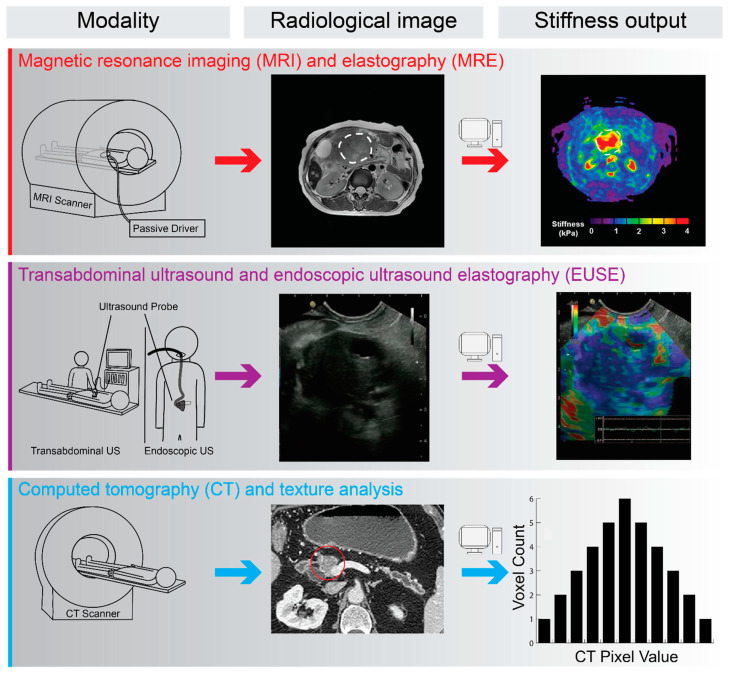
Schematic illustration of radiological methods used in the diagnosis of PDAC with the potential for biomechanical assessment of tissue properties. For each method, the radiological output and post-processed mechanical properties are presented. These non-invasive measures of tissue mechanics may provide a surrogate measurement for PDAC tissue stiffness, with the potential to predict patient outcomes and inform treatment options. The passive driver illustrated in the MRI schema provides vibrational input for magnetic resonance elastography (MRE). MRE scan and output images reproduced with permission from An et al., *Clin. Radiol*. **2016**, *71*, 1068.e7–1068.e12; published by Elsevier Ltd. [113]. EUSE images reproduced with permission from Cui et al., *World J. Gastroenterol*. **2015**, *21*, 13212-13224; published by Baishideng Publishing Group [114]. CT pancreas image reproduced with permission from Ren et al., *Front. Oncol*. **2019**, *9*, 1171; published by Frontiers Media SA [115].

Given the growing recognition of the role played by tissue stiffness in PDAC progression, it may be of great benefit to quantify PDAC biomechanics as part of the diagnostic pathway. Radiological methods facilitate this in a non-invasive manner. Here, we review the imaging modalities that are most used in the diagnosis of pancreatic cancer [110,111,112] (Figure 3).

Magnetic resonance elastography (MRE) provides one such method. This imaging method works on the principle of biophysically perturbing tissue using harmonic excitation and measuring the resultant strain waves over tens of mm. Local shear wave displacement patterns are imaged and with post-processing provide for the generation of elastograms to visually represent tissue stiffness [116]. This technique has been used with great success in the assessment of liver fibrosis [117,118]. Elastography is more challenging for pancreatic tissue than that of liver, especially in the case of PDAC, due to the heterogenous tissue and deep location within the abdomen [113,119]; however, it has been shown to yield reproducible results in healthy pancreata and cancerous tissue [113,119]. MRE measurements, namely the stiffness ratio (stiffness of the mass divided by that of the parenchyma), have been observed to be superior to CA19-9 (cancer antigen 19-9) for the assessment of benign versus malign pancreatic lesions [120]. This is consistent with other studies reporting MRE’s ability to differentiate PDAC and benign pancreatic tumours [121]. However, it should be noted that MRE is best used to complement traditional imaging techniques rather than replace them, and concerns have been raised over MRE’s ability to accurately detect stiffness values in smaller tumours.

Tissue stiffness can also be assessed using endoscopic ultrasound elastography (EUSE). While a relatively invasive method, this is particularly relevant as standard endoscopic ultrasound is commonly used to guide fine needle sampling of tissue in suspected pancreatic malignancy [122]. EUSE works on the premise that tissue stiffness is recorded through physical compression with the ultrasound probe [123]. Vibrations are provided in a physiological manner, i.e., through blood vessel pulsations and respiration. Small structural deformations within the B-mode ultrasound image are captured with the degree of deformation used as an indicator of tissue stiffness [124]. Both qualitative and quantitative assessment of tissue is possible with relative elasticity presented qualitatively using colour map of relative stiffness (Figure 3). Relative stiffness can be quantified within regions of interest to generate a hue histogram. The strain ratio between the target lesion and a reference soft region is another quantitative measure [125]. A meta-analysis of EUSE in differentiating PDAC from inflammatory masses showed promising results with high sensitivity, but only a moderate specificity [126]. While several other studies also demonstrate the ability of EUSE to detect PDAC as a mass of increased stiffness in vivo [124,125,127,128], sensitivity of EUSE is an issue and it is not ready to replace the biopsy in PDAC diagnosis. However, it could provide a useful adjunct to biopsy sampling and can also provide improved guidance to the biopsy site [128]. Given the inverse relationship between stiffness and drug delivery, it has been suggested that elastography be used to predict drug penetration in PDAC [129]. A further application may lie in evaluating lesions pre- and post-NAT as a prediction of response and outcome.

A non-endoscopic ultrasound elastography technique is shear wave elastography (SWE) which includes transient elastography (Fibroscan, Echosens), point SWE, 2D- and 3D-SWE (e.g., Virtual Touch Quantification (VTq), Siemens). VTq, also termed acoustic radiation force impulse elastography, has been used with success in the evaluation of liver fibrosis [130]. It estimates shear wave velocity and translates this into tissue stiffness. It has demonstrated use in PDAC, with PDAC tissue having higher shear wave velocity values than normal pancreatic tissue, and a negative predictive value of 95% [131]. It is not applied to endoscopic ultrasound which may limit its predictive use, but also is a less invasive procedure. SWE using FibroScan has also been shown to correlate well to liver stiffness in the diagnosis of non-alcoholic fatty liver disease (NAFLD) [132,133] and has recently been demonstrated to predict survival in chronic liver disease [134].

Another ultrasound technique, harmonic motion imaging (HMI), has recently been shown to enable detection of mouse PDAC versus inflamed pancreatic tissue. It has been suggested this technique could be incorporated into transabdominal or endoscopic ultrasound examinations [135]. It overcomes many limitations of other techniques by using two transducers, one to perturb the tissue, the other to image, making it possible to reach deep organs such as the pancreas [136]. In addition, harmonic motion imaging could detect a decrease in stiffness post NAT in patient tissue [135]. Harmonic motion elastography (HME) confirmed these findings [91], and can be used to delineate between PDAC and non-neoplastic pancreas to delineate the tumour margin through stiffness assessment. HMI and HME differ in that HMI measures oscillatory displacement while HME measures filtered shear wave data [91].

While not a direct measure of tissue stiffness, CT texture analysis may provide a measure of PDAC stromal organisation as a proxy for PDAC stiffness. CT texture analysis provides additional detail further to what can be seen on radiological imaging by examining the values of the image pixels and applying statistical, transform or model-based algorithms to said values. A simple example of an output produced by texture analysis could be the grey-value distribution within an image [137] (Figure 3). This method has been successfully employed in the measurement of hepatic fibrosis although further refinements to the modality are warranted [138]. Another study compared CT and MRI texture analysis in liver fibrosis and rated MRI as more accurate, however, no method was recommended for clinical use [139]. In contrast, Budai et al. successfully differentiated high- and low-grade liver fibrosis using texture analysis applied to CT imaging [140]. This approach may be more feasible in many settings due to the wider availability of CT scanners comped to MRI and the relatively operator independent nature of CT and MRI, which is not the case for the US. A relevant proof of concept study has been undertaken in pancreatic tissue by Kambakamba et al. who demonstrated the ability of machine learning with texture analysis to recognise features of pancreatic pathology and predict outcome, warranting future prospective analysis [141]. Furthermore, CT analysis has the benefit of enabling correlation with other in vitro methods of measurement, as well as the analysis of NAT and post resection specimens.

## 5. Future Research Perspectives

There is a growing appreciation for the role of tissue mechanics in the progression of PDAC, evidenced by the growing attention to biomechanics in mouse model and in vitro studies. However, given the clear differences between species, there remains a requirement for further biomechanical analysis of surgical specimens throughout PDAC progression. These specimens are difficult to obtain but will provide a greater understanding of the role of mechanics, matrix abundance and heterogeneity in the evolution of PDAC. As discussed, evolving stiffness is thought to prevent drug delivery and support chemoresistance through several routes, which highlights the need to further understand the link between tumour mechanics, treatment options and prognosis.

The heterogeneous nature of PDAC tissue is a known driver of aggression with the level of differentiation providing a key diagnostic statistic. The periductal region surrounding tumour glands has been found to have increased stiffness which drives metastasis [57]. The use of pre-clinical imaging to provide a measure of tissue heterogeneity could provide for greater specificity in diagnosis. Heterogeneity also poses the risk of obtaining inconsistent results and reinforces the importance of having a standardised sampling technique.

Other relevant directions for future research and innovation include the use of radiomics to characterise pancreatic tumours in situ and correlate with outcomes following intercurrent treatment. There will also likely be a significant increase in the use of artificial intelligence and machine learning to gain insights into these radiomics based on stiffness characteristics to inform risk stratification going forward [142,143,144].

Evaluation of biopsy and resection specimens using the in vitro techniques outlined above and correlating with clinically relevant outcomes has enormous potential in aiding our understanding of tumour stiffness in PDAC. These data can also be combined with both computational models to better understand the role of stiffness in tumour cell behaviour and to train machine learning datasets for radiomics analysis. There is a paucity of human studies to date, but with the evolution and increasing efficiency and value of these techniques, they will likely become more popular. Combining these techniques with clinically relevant data in the setting of NAT, has enormous potential.

## 6. Conclusions

It is recognised that studies show that the stiffness of PDAC is linked with increased invasive potential of the tumour and decreased drug delivery, leading to worsened patient outcomes. Some studies report that decreasing the stiffness of the tumour also leads to worsened outcomes, indicating more research is required to deepen our understanding of the mechanisms at play. There are several testing options available for the characterisation of PDAC biomechanics ex vivo and in vivo which have been discussed herein. The authors believe it to be prudent to recommend further mechanical testing of PDAC surgical specimens, with and without NAT, to observe the effect of the mechanical properties of the tissue and what this might mean for patient outcomes.

## Figures and Tables

**Table 1:** Terms used to describe PDAC biomechanics.

Term	Definition
Mechanical properties	Physical properties of a material under force.
Mechanical stress	Force per unit area due to the action of a force, can be either compressive, tensile or shear in action. Unit: Pa or mmHg
Solid stress	Mechanical forces contained in and transmitted by solid and elastic elements of the extracellular matrix and cells. Unit: Pa or mmHg.
Interstitial fluid pressure	Pressurisation of interstitial fluid. Unit: Pa.
Stiffness (elasticity)	Resistance of a material to load. Calculated as stress per unit strain. Young’s modulus (E) used to describe elastic materials. Storage (G’) and Loss (G’’) modulus used to describe viscous materials. Unit: Pa.
Compliance	Inverse of stiffness. Unit: Pa^−1^
Viscoelasticity	Term used to describe materials with both viscous and elastic characteristics resulting in time-dependent strain under load.
